# Anæsthetics

**Published:** 1874-05

**Authors:** 


HALL'S

### JOURNAL OF HEALTH

AND

MISCELLANY.

VOL. XXI.—MAY, 1874.—NO. V.

### ANÆSTHETICS.

ANÆSTHESIA means "without feeling." It is applied now to insensibility to pain by breathing certain kinds of air or odors for the purpose of undergoing operations without feeling the pain of them. These are nitrous oxyde, ether and chloroform. The first, or laughing gas, is entirely safe, and is breathed pure, wholly without any admixture of common air. Chloroform is the most dangerous; it should not be breathed unless there is ninety-seven per cent. of air mixed with it. In breathing ether, the throat should be gradually accustomed to it by sniffing it for a few breaths, and then exclude the air altogether. General readers should acquaint themselves with a few facts about the nature, uses and effects of anæsthetics. No one should use them who has any form of heart or lung disease. It is better not to take them at all, in view of an operation, unless the system is fully under its power.

There is danger if, while a person is breathing an anæsthetic, the inspiration is hard and short, or the expiration feeble and long, or the chest ceases to move, or snoring in the throat. Only chloroform induces an arrest of the heart's action; then the danger of death is most imminent. The instant the chest becomes still in animals or man, forced breathing must be induced by blowing air down the windpipe with the mouth or bellows, or death will ensue. It is safer to rely on the motion of the chest than on the pulse, for, if the pulse stops, it is death; but, for some time after breathing ceases, the pulse still beats, and there is possibility of resuscitation. There is danger if the skin begins to turn black, while great paleness of the face indicates that the heart is about ceasing to act, and death approaches. Death by anæsthetics is a suffocation, just as in drowning, the first effect of which is want of sensation or feeling; then the side of the brain is paralyzed, and we become unconscious; next the back part of the brain becomes deadened, and muscular action ceases; then the spinal cord is put to sleep, and there is no voluntary motion; and, last of all, the medulla oblongata, the connecting part between the brain and the spinal cord, that part of the nervous system which presides over the muscles of involuntary motion, as the heart, is paralyzed, the pulse ceases to beat, and the man dies.

A man may be so drunk as to be without feeling, with no sense of pain.

The ancients knew several substances which destroyed sensation—that is, induced inaction, paralysis of the nerves of sensation. The Greeks and Romans used mandragora, a narcotic plant, known as "mandrake," to deaden the pain of surgical operations. So did the Chinese, but not until modern times has any attempt been made to deaden sensation by inhalation. The inhalation of sulphuric ether was used to mitigate asthma in 1795; as early as 1822 it was ascertained that it would cause asthma, and what causes a disease can cure it, according to Homœopathy; but it is only within thirty years that inhalation has been used with the express purpose of causing insensibility to the pain of a contemplated surgical operation. The first was in reference to tooth drawing, tried on his own person by Horace Wells, of New Haven, some months before, and Dr. Morton, of Boston, later on.

